# Establishment of a zebrafish hematological disease model induced by 1,4-benzoquinone

**DOI:** 10.1242/dmm.037903

**Published:** 2019-03-28

**Authors:** Ao Zhang, Mei Wu, Junliang Tan, Ning Yu, Mengchang Xu, Xutong Yu, Wei Liu, Yiyue Zhang

**Affiliations:** 1 Division of Cell, Developmental and Integrative Biology, School of Medicine, South China University of Technology, Guangzhou 510006, China; 2 Key Laboratory of Zebrafish Modeling and Drug Screening for Human Diseases of Guangdong Higher Education Institutes, Department of Developmental Biology, School of Basic Medical Sciences, Southern Medical University, Guangzhou 510515, China

**Keywords:** 1,4-Benzoquinone, Hematotoxicity, Neutrophilia, *c-myb*, Zebrafish

## Abstract

Benzene exposure is associated with various hematological disorders, in particular leukemia. The reactive metabolite of benzene, 1,4-benzoquinone (BQ), generated in bone marrow, is suggested to be a key molecule in mediating benzene-induced hematotoxicity and carcinogenicity. However, its pathogenic role remains largely unknown due to a lack of suitable vertebrate whole-organism models. Here, we present an *in vivo* study to reveal the effect of BQ exposure on hematotoxicity in zebrafish. From embryonic stages to adulthood, BQ exposure suppressed erythroid and lymphoid hematopoiesis but led to abnormal accumulation of myeloid cells and precursors, which resembles benzene-induced cytopenia and myeloid dysplasia in humans. This myeloid expansion is caused by granulocyte, but not macrophage, lineage, emphasizing the significant role of lineage specificity in BQ-mediated hematopoietic toxicity. Analysis of the *c-myb* (also known as *myb*)*-*deficient mutant *cmyb^hkz3^* revealed that BQ induced neutrophilia in a *c-myb*-dependent manner, demonstrating that *c-myb* is a key intrinsic mediator of BQ hematotoxicity. Our study reveals that BQ causes lineage-specific hematotoxicity in zebrafish from embryonic stages to adulthood. Since *c-myb* is indispensable for BQ to induce neutrophilia, *c-myb* could serve as a potential drug target for reversing BQ hematotoxicity.

## Abstract (precis)

**Summary:** Acute exposure to 1,4-benzoquinone leads to lineage-specific hematotoxicity in zebrafish from embryonic stages to adulthood, resembling benzene-induced cytopenia and myeloid dysplasia in humans.

## INTRODUCTION

For decades, epidemiological studies have found that occupational and environmental exposure to benzene can have harmful effects on the immunological, neurological, reproductive and, especially, hematological systems, causing cytopenia, aplastic anemia and leukemia (Wilbur et al., 2007). Benzene is classified as a Group 1 carcinogen in humans and animals (IARC, 1982). Benzene metabolism occurs principally in the liver and lungs, with secondary metabolism in bone marrow (BM) (McHale et al., 2012). 1,4-Benzoquinone (BQ) is generated by benzene in the BM, and it causes damage by forming protein and DNA adducts, and producing reactive oxygen species (ROS) (Snyder and Hedli, 1996; Wan and Winn, 2007), Subsequently, excess ROS induce oxidative stress and promote apoptosis (Circu and Aw, 2010). It has been suggested that BQ plays crucial roles in mediating benzene-induced hematological toxicity and carcinogenicity. In support of this, previous studies have demonstrated that the BQ-detoxifying enzyme NAD(P)H: quinone oxidoreductase 1, mutation of which is associated with human susceptibility to benzene hematological toxicity (Rothman et al., 1997), can protect mice against benzene-induced myelodysplasia (Long et al., 2002; Iskander and Jaiswal, 2005). Previous *in vitro* studies have shown that exposure of murine hematopoietic stem and progenitor cells (HSPCs) to BQ interferes with their physiological properties and changes their clonogenic potency by altering genes for apoptosis, DNA repair, cell cycle, self-renewal and differentiation (Chow et al., 2018; Faiola et al., 2004). Son et al. (2016) analyzed the impact that BQ exposure has on DNA-repair-defective mouse embryonic stem cells. They proposed that BQ suppresses type 1 topoisomerases to inhibit replication fork restart and progression, leading to chromosomal instability that has the potential to cause hematopoietic disorders. To date, there is no vertebrate model for hematological toxicity of BQ exposure, which limits the investigation at the whole-organism level.

Although BQ is known to be involved in hematological toxicity and cancer, how embryonic and adult hematopoiesis are affected and the molecular and cellular bases have not been fully elucidated. BQ metabolism and its molecular mechanisms have been studied for several decades, but animal models for evaluating the hematological effects of BQ from embryonic stages to adulthood are still lacking. The zebrafish is an effective vertebrate model for studying hematopoiesis *in vivo* and for investigating the pathogenesis of hematological disorders, owing to high fecundity, optical transparency and highly conserved hematopoiesis (Rasighaemi et al., 2015). In this study, we investigated the hematotoxicity of BQ in a zebrafish model, and found that the BQ-treated zebrafish exhibited cytopenia and myeloid dysplasia, which resembled benzene-induced hematotoxicity in mammals.

## RESULTS

### Embryotoxicity and teratogenicity of BQ in zebrafish

**Fig. 1.**
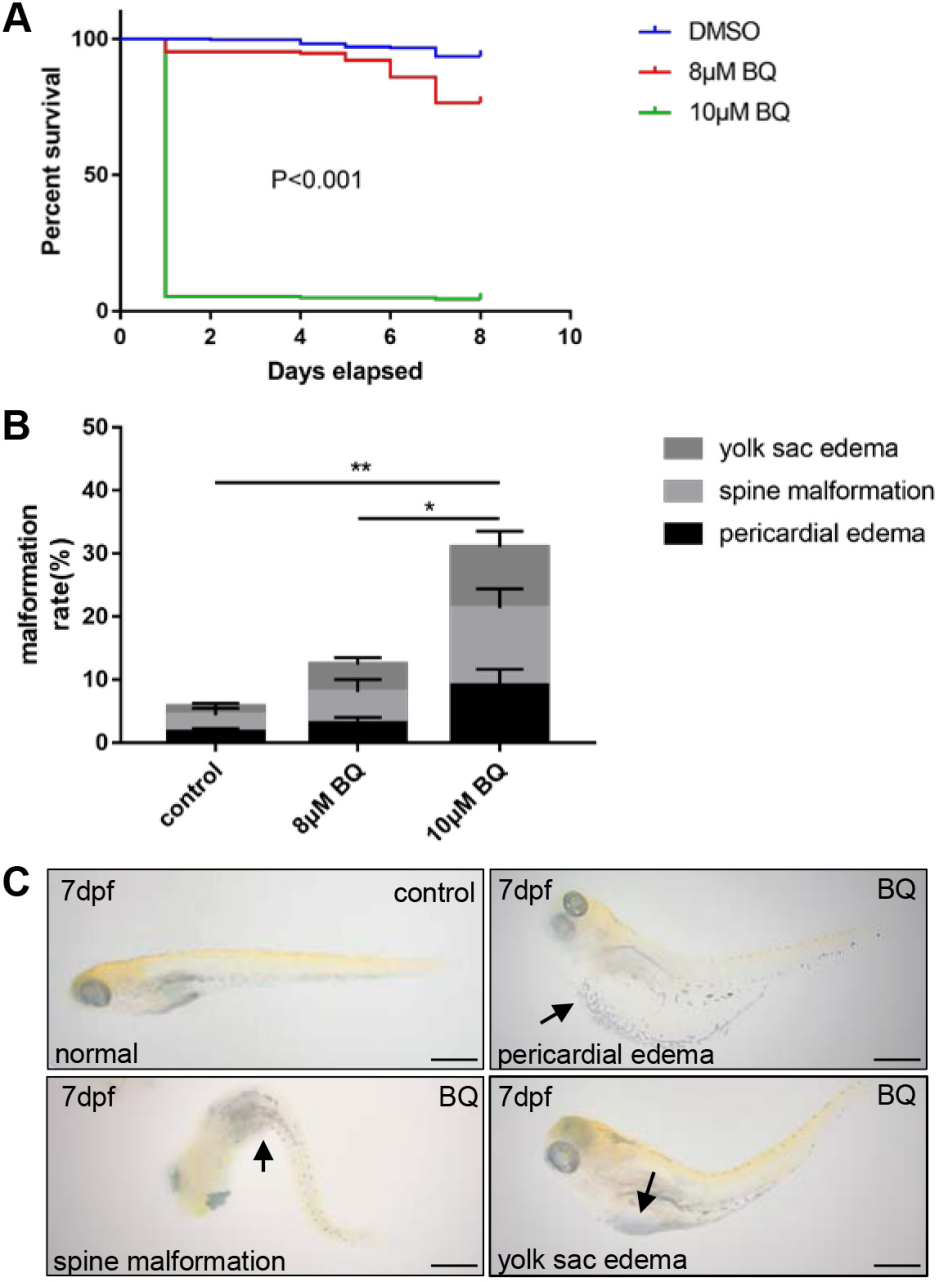
**Kaplan–Meier survival curve and analysis of zebrafish embryo phenotype following exposure to 1,4-benzoquinone (BQ).** (A) Survival rates in zebrafish embryos following continuous exposure to BQ (*n*=278 per group) in the range 8–10 μM, from 2 dpf to 9 dpf (log-rank test, *P*<0.001). (B) Phenotypic traits (indicated by the arrows in C) observed following BQ exposure (Chi-squared test, mean±s.d., *P<0.05, **P<0.01). (C) Typical malformations were observed in zebrafish embryos exposed to BQ. Scale bars: 500 μm.

To investigate the effect of BQ on hematopoiesis, zebrafish embryos were treated with BQ. We first determined the embryotoxicity and teratogenicity of BQ by recording the survival and malformation rates every 24 h after BQ exposure. Absence of swim activity, heart beat and tail blood flow were used as criteria to differentiate a viable from a non-viable larval zebrafish. The Kaplan–Meier curve showed that, at 7 days post-fertilization (dpf), recorded survival rates were 94% and 77% in the control and 8 μM BQ groups, respectively. The survival rate was markedly decreased as the concentration of BQ increased to 10 μM (Fig. 1A). Malformations, such as yolk sac edema, spine malformation and pericardial edema, were occasionally observed in developing embryos (∼6%) (Fig. 1B,C). However, BQ exposure resulted in a dose-dependent increase in malformation rate, ∼13% and ∼31% in 8 μM BQ and 10 μM BQ groups, respectively, but the percentages of each malformation at all three doses were not significantly different (Fig. 1B). The above data demonstrated the fetal toxicity of BQ. Thus, the concentration of 8 μM BQ was chosen in subsequent experiments to evaluate BQ hematotoxicity in zebrafish.

### BQ exposure results in abnormal hematopoiesis in zebrafish embryos

**Fig. 2.**
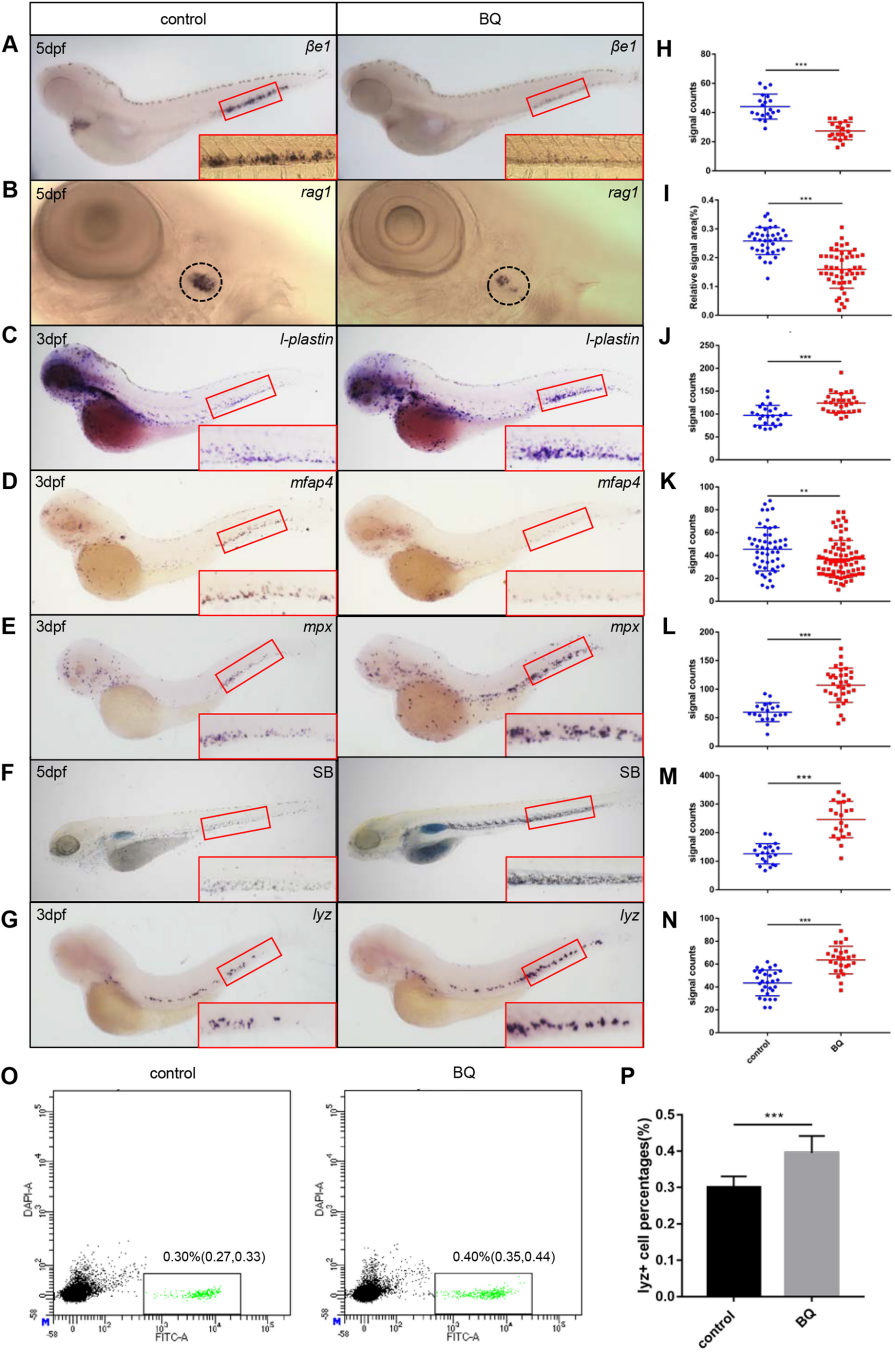
**Expression of lineage-specific markers in zebrafish was detected using WISH and SB staining.** (A,B) Loss of *βe1-globin* (*n*=20) and *rag1* (control group *n*=37, BQ group *n*=49) expression in 5 dpf embryos exposed to BQ. The red boxed regions show magnified views of the caudal hematopoietic tissue (CHT). Relative *rag1^+^* thymocyte-signal areas were analyzed using ImageJ software. The circled regions show the thymus. (C) Increase in *l-plastin* expression upon BQ exposure at 3 dpf (control group *n*=25, BQ group *n*=30). (D) Expression of the macrophage marker *mfap4* was reduced in the BQ exposure group (control group *n*=52, BQ group *n*=76). (E,G) The neutrophil markers *mpx* (E) (control group *n*=20, BQ group *n*=33) and *lyz* (G) (control group *n*=39, BQ group *n*=35) both showed increased expression in embryos at 3 dpf following BQ exposure. (F) Increased SB^+^ cells were observed in the BQ exposure group compared with the control group (*n*=21 per group). (H–N) Quantification of WISH and SB staining for A–E (Student's *t*-test, mean±s.d., **P<0.01, ***P<0.001). (O) Dot plot of flow cytometry analysis of *lyz:*GFP^+^ cells from control (left) and BQ-treated fish (right). Three independent experiments (in each group, 100 embryos are pooled together) were conducted. (P) Percentage of *lyz*^+^ cells in each group [0.30% in the control group and 0.40% in the BQ group; Chi-squared test (95% c.i.), ***P<0.001].

Occupational and environmental exposure to benzene is associated with the incidence of hematological disorders and malignancies, such as aplastic anemia, myelodysplastic syndrome (MDS) and acute myeloid leukemia (AML) (Baan et al., 2009; Linet et al., 2015; McHale et al., 2012), as well as MDS progression to AML (Greenberg et al., 2012; Poynter et al., 2017; Schnatter et al., 2012). To explore the hematotoxicity of BQ, we monitored zebrafish embryonic hematopoiesis by performing whole-mount *in situ* hybridization (WISH) of lineage specific markers in 3 dpf and 5 dpf larval zebrafish. Expression of the erythroid marker, *βe1-globin* (also known as *hbbe1.1*) (Paffett-Lugassy et al., 2007), was decreased in BQ-exposed embryos (Fig. 2A,H). Likewise, expression of the lymphoid marker *rag1* (Willett et al., 1997) was also reduced (Fig. 2B,I). These data indicate that BQ may lead to deficiencies of erythrocytes and lymphocytes, which is consistent with cytopenia and anemia in patients exposed to benzene (Aksoy, 1989). For myeloid lineage, expression of *l-plastin* (Herbomel et al., 2001), a marker for both neutrophils and macrophages, was significantly increased in the BQ-treated embryos (Fig. 2C,J). To unveil whether the myeloid expansion was caused by granulocyte or macrophage lineage cells, we checked the neutrophil and macrophage markers, respectively. Expression of the macrophage lineage marker *mfap4* (Zakrzewska et al., 2010) was decreased in BQ-treated embryos (Fig. 2D,K). However, expression of the granulocytic marker *mpx* (Lieschke et al., 2001) (Fig. 2E,L) and Sudan Black B (SB) staining (Le Guyader et al., 2008) (Fig. 2F,M) were significantly elevated after BQ exposure, suggesting robust expansion of granulocytic lineage cells in BQ-exposed embryos. We also found that another granulocytic marker, *lyz* (Liu and Wen, 2002), was increased as well (Fig. 2G,N), which was further supported by flow cytometry analysis of *Tg(lyz:GFP)* embryos (Fig. 2O,P). These data demonstrate that BQ causes anemia and neutrophilia in zebrafish embryos, which resembles the hematotoxicity of benzene in mammals (Snyder and Kocsis, 1975).

### BQ exposure causes neutrophilia due to accelerated neutrophil proliferation

**Fig. 3.**
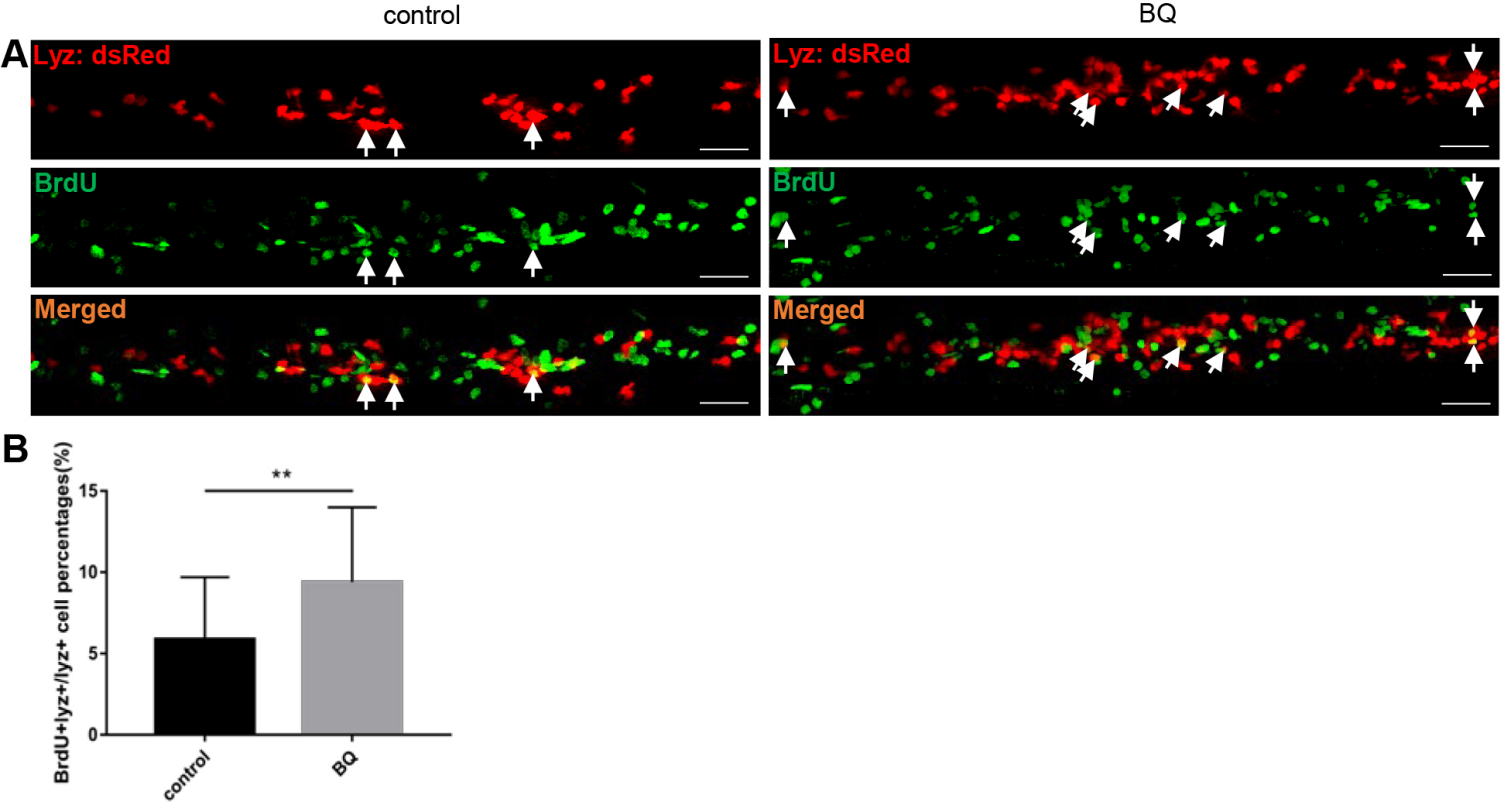
**Exposure to BQ induced neutrophil proliferation in zebrafish embryos.** (A,B) BrdU incorporation assay. Double staining of BrdU/*lyz* (A) showed BrdU incorporation of CHT *lyz*^+^ cells in BQ-exposed embryos and controls at 3 dpf. Arrows indicate *lyz*/BrdU double-positive cells. Scale bars: 50 μm. Percentage of the CHT-localized *lyz*^+^ myeloid cells that incorporate BrdU (B) in *lyz*^+^ myeloid cells (Student's *t*-test, control group *n*=30, BQ group *n*=26, mean±s.d., **P<0.01).

To establish whether the increase in neutrophils after BQ exposure is due to accelerated cell proliferation, we performed a bromodeoxyuridine (BrdU) incorporation assay, which is commonly used for detection of proliferating cells in living tissues (Lehner et al., 2011). Embryos of the neutrophil-specific transgenic line *Tg(lyz:dsRed)* were exposed to BQ and subjected to BrdU incorporation assay at 3 dpf. The proportion of BrdU and *lyz:dsRed* double-positive neutrophils among total *lyz:dsRed*^+^ neutrophils in the caudal hematopoietic tissue (CHT) region was increased in the BQ-treated compared with control group (Fig. 3A,B), indicating that neutrophil proliferation was accelerated by BQ.

### BQ promotes neutrophilia through *c-myb*

**Fig. 4.**
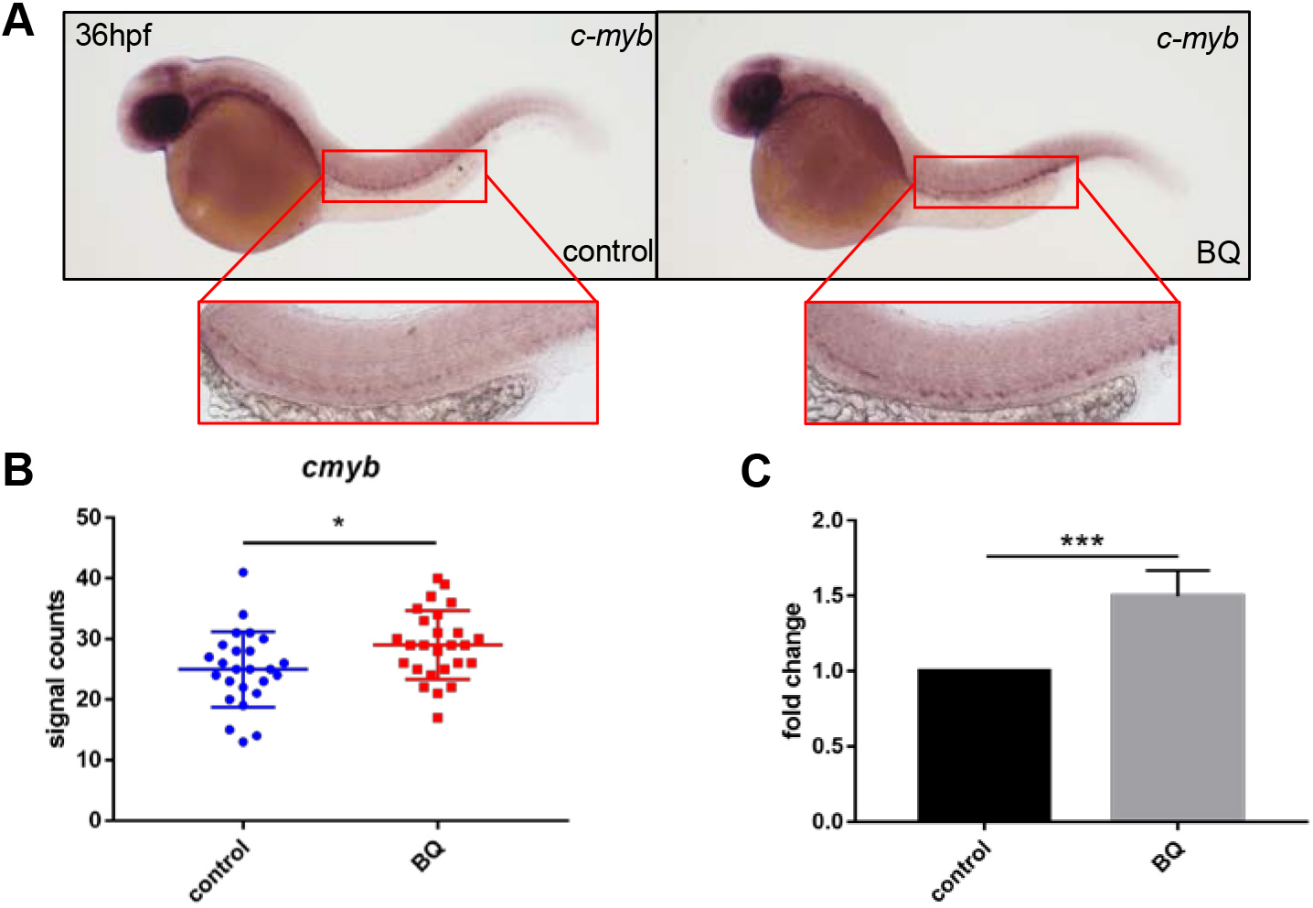
**Effect of BQ exposure on *c-myb* expression.** (A–C) BQ exposure increased *c-myb* expression. Both WISH performed at 36 hpf (A,B) and RT-qPCR performed at 2 dpf (C) indicated increased expression of *c-myb* in BQ-exposed (right, A) and control (left, A) groups (Student's *t*-test, *n*=25 or 26, mean±s.d., *P<0.05, ***P<0.001)*.* The red boxed regions show magnified views of the aorta-gonad-mesonephros (AGM).

**Fig. 5.**
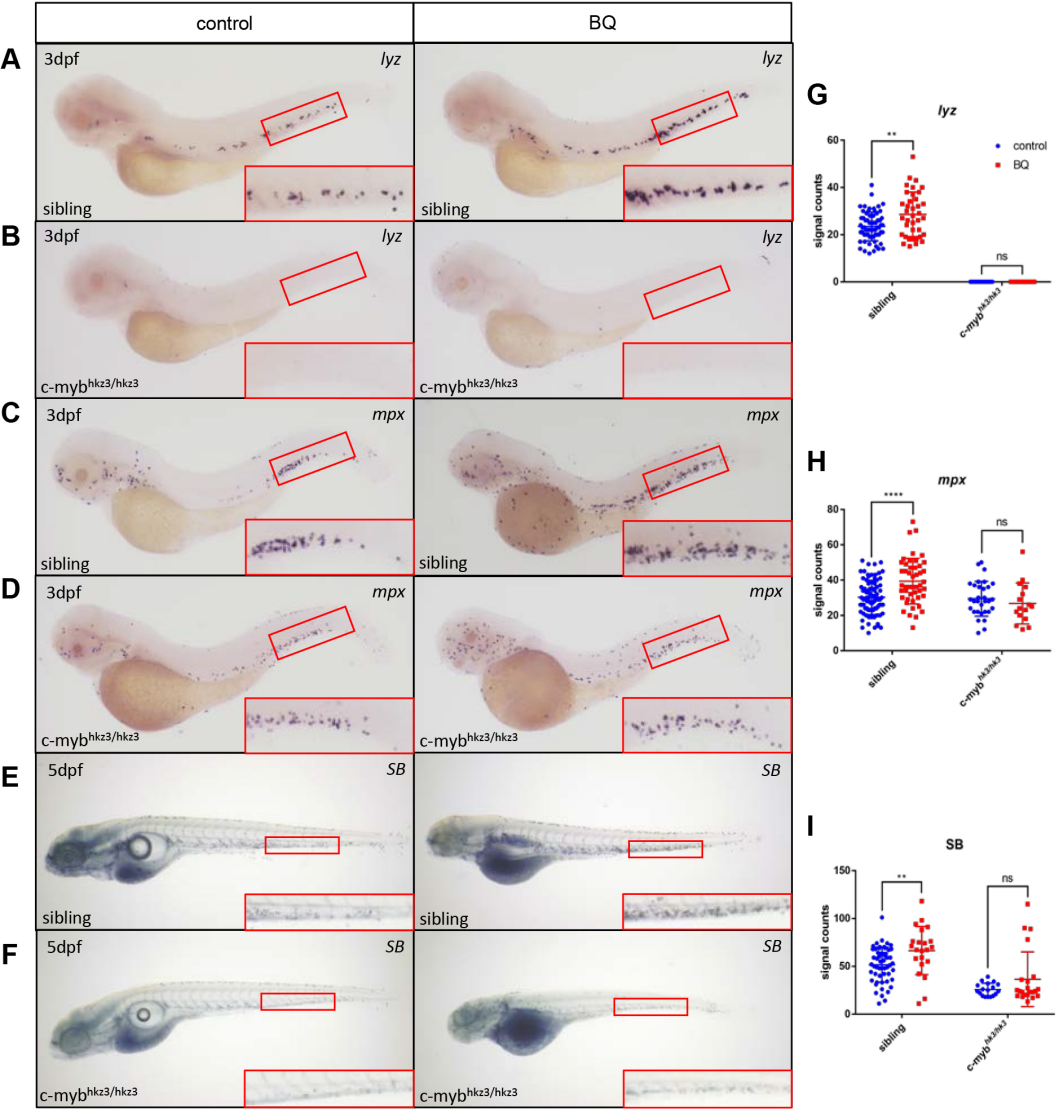
**BQ-induced neutrophil expansion was blocked in *c-myb*-deficient mutants.** (A–I) Expression of the neutrophil markers *lyz* (A,B) (sibling control group *n*=65, BQ group *n*=38; mutant control group *n*=25, BQ group *n*=35) and *mpx* (C,D) (sibling control group *n*=83, BQ group *n*=47; mutant control group *n*=31, BQ group *n*=16) was analyzed using WISH, and SB staining (E,F) (sibling control group *n*=54, BQ group *n*=18; mutant control group *n*=25, BQ group *n*=22) was detected, in *c-myb^hkz3/+^* intercrossed progenies in BQ-treated groups (right column) and untreated controls (left column). Signals increased following BQ exposure at 3 dpf in siblings (A,C,E) but were unaltered in *c-myb^hkz3/hkz3^* mutants (B,D,F). The red boxed regions show magnified views of the CHT. (G–I) Quantification of WISH and SB staining for panels A–F (Student's *t*-test, mean±s.d., **P<0.01, ****P<0.0001; ns, non-significant).

The proto-oncogene *c-myb* (also known as *myb*) is a key regulator of hematopoietic cell proliferation and differentiation, and correct levels of *c-myb* are essential for regulating distinct differentiation steps during hematopoietic cell development, as well as in leukemogenesis (Sakamoto et al., 2006; Wolff, 1996). Our previous study in zebrafish showed that *c-myb* is essential for neutrophil differentiation (Jin et al., 2016), and *c-myb* activation causes MDS-like phenotypes due to enhanced myeloid cell proliferation (Liu et al., 2017). It has been shown that exposure to BQ results in increased *c-myb* transcriptional activity and phosphorylation of c-Myb in cell lines (Singh and Winn, 2008; Wan et al., 2005), although *in vivo* evidence is still required. We monitored whether *c-myb* expression was altered upon BQ exposure in zebrafish. By performing WISH and reverse transcription quantitative polymerase chain reaction (RT-qPCR), we found that, in BQ-treated embryos, *c-myb^+^* signals were significantly elevated in the aorta-gonad-mesonephros (AGM) region (Fig. 4A,B), and *c-myb* expression was increased as well (Fig. 4C). To examine whether *c-myb* is a mediator for BQ-induced neutrophilia, we treated null *c-myb^hkz3/hkz3^* mutants with BQ (Jin et al., 2016). Neutrophil-specific markers (*lyz*, *mpx* and SB staining) were elevated in siblings after BQ exposure (Fig. 5A,C,E), as described in wild-type embryos (Fig. 2D–F). However, neutrophils were not further induced by BQ when *c-myb* was absent, as demonstrated by the unaltered neutrophil markers with or without BQ treatment in *c-myb^hkz3/hkz3^* mutants (Fig. 5B,D,F). These data suggest that *c-myb* is required for BQ-induced neutrophilia, and that it functions as a key mediator for BQ hematotoxicity *in vivo*.

### BQ exposure causes myeloid dysplasia in adult fish

**Fig. 6.**
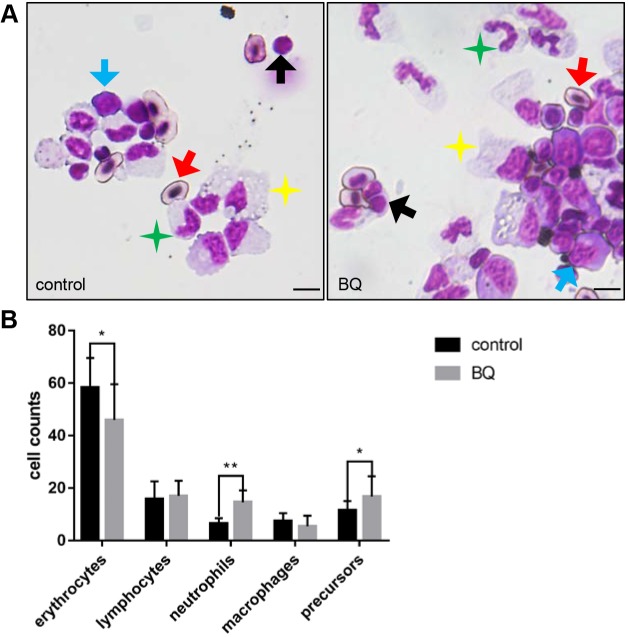
**BQ hematotoxicity to adult fish.** (A) May–Grunwald/Giemsa of KM cells in BQ-exposed (right) and control (left) fish. Red arrows, erythrocytes (oval-shaped nucleus and cytoplasm); black arrows, lymphocytes (large round-shaped nucleus surrounded by minimal cytoplasm); green stars, neutrophils (segmented nucleus and cytoplasm with distinct neutral granules); yellow stars, macrophages (large irregular-shaped cytoplasm with distinct vacuoles); blue arrows, precursors (large and dense nucleus with dark cytoplasm). Scale bars: 5 μm. (B) Blood cell counts of KM in each group were calculated manually based on their morphology (Student's *t*-test, *n*=9, mean±s.d., *P<0.05, **P<0.01).

To explore whether adult hematopoiesis could be affected by BQ, we further investigated the cytological changes in myelogram of adult zebrafish following BQ exposure. Kidney marrow (KM) cells were collected from adult fish in the control and BQ exposure groups for cytological analysis and blood cell count. In BQ-exposed fish KM, the proportion of erythrocytes decreased, whereas the proportions of neutrophils and precursors were significantly increased (Fig. 6A,B). Exposure to benzene can lead to leukemia in humans, especially myeloid leukemia (Schnatter et al., 2012). The increase in hematopoietic precursors in the KM of BQ-treated fish resembled the hematopoietic disorders in benzene-exposed mice and humans, which suggested a tendency to progress to hematopoietic malignancy.

## DISCUSSION

BQ is a reactive metabolite of benzene that is produced in BM. It is believed to be mainly responsible for the myelotoxicity/myeloid neoplasms observed in the BM of people exposed to benzene (Hartwig, 2010). To establish the mechanism linking benzene exposure to hematotoxicity and carcinogenicity at the whole-organism level, we explored the effects of BQ on hematopoiesis and hematological changes in zebrafish. We showed that exposure to BQ increased the mortality and malformation rate in a dose-dependent manner, and the rate of malformations in multiple organs, suggesting BQ embryotoxicity in zebrafish. We demonstrated that BQ caused hematopoiesis perturbation from embryonic stages to adulthood, resembling human hematological diseases. At a molecular level, we uncovered *c-myb* as a key mediator of BQ-induced neutrophilia. Our study implies that *c-myb* serves as a molecular marker for evaluation of hematotoxicity upon BQ exposure, and that it could serve as a potential drug target for reversing BQ hematotoxicity.

We found that non-lethal doses of BQ impaired erythroid and lymphoid hematopoiesis and abnormally increased neutrophils. The myeloid dysplasia could be partially interpreted by the acceleration of myeloid cell proliferation upon BQ exposure. Likewise, in adult fish, BQ exposure also resulted in reduction in other blood cell lineages but an increase in neutrophils, while the proportion of precursors increased in KM as well, similar to the benzene-induced cytopenia and myeloid dysplasia in humans. Our data suggest that, from embryonic stages to adulthood, BQ induces abnormal expansion of myeloid cells, which may increase the risk of myeloid leukemia. However, more studies are required to confirm whether the duration of BQ treatment is long enough to induce hematological malignancies.

For the molecular and cellular mechanisms underlying BQ hematotoxicity, several molecular pathways have been proposed, such as type 1 topoisomerases (Son et al., 2016). It has been shown that BQ modulates the fate of HSPCs by altering the self-renewal- and differentiation-related genes, such as *Bmi-1* and *GATA3* (Chow et al., 2018). We found that *c-myb* is a key mediator in the effect of BQ on neutrophil expansion. How these molecules and pathways crosstalk upon BQ exposure requires further elucidation, which will help us to understand how benzene and BQ mediate leukemogenesis.

Clinically, *c-myb* is highly expressed in leukemic cells in patients with AML, chronic myeloid leukemia (CML) and acute lymphoblastic leukemia (Machov; Polakov; et al., 2011; Siegert et al., 1990). It is essential for the proliferation and maintenance of leukemic cells, and aberrant *c-myb* activity is associated with human myeloid leukemia (Westin et al., 1982). High expression of *c-myb* is believed to be associated with oncogenic activity and poor prognosis in human AML (Gopal et al., 1992). Our previous study revealed that zebrafish with *c-myb^hyper^* (*c-myb* with hyperactivity) display MDS-like phenotypes from embryonic stages, and can develop myeloid and lymphoid leukemia-like phenotypes in adulthood (Liu et al., 2017). In this study, BQ caused myeloid cell expansion in neutrophil, but not macrophage, lineage cells, which is similar to the hematopoietic phenotype in *c-myb^hyper^* zebrafish. Consistently, the upregulated *c-myb* level upon BQ treatment suggests that BQ leads to hematological disorders via *c-myb*. In the absence of *c-myb*, BQ had no effect on neutrophils, implying that BQ hematotoxicity could, at least partially, be reversed by targeting *c-myb*.

In summary, we generated an animal model for *in vivo* investigation of the hematotoxicity of BQ exposure. Our data indicate that BQ exposure promoted proliferation of myeloid cells but suppressed other blood lineage cells in a zebrafish model, which shares similar pathological features with myeloid dysplasia in humans. At a molecular level, *c-myb* is a key factor in the response to BQ-mediated hematotoxicity, and it could serve as a potential target for preventing or reversing BQ or benzene hematotoxicity.

## MATERIALS AND METHODS

### Zebrafish husbandry

All experiments were performed according to the guidelines laid down by the Institutional Animal Care and Use Committee of Southern Medical University and the South China University of Technology. The following strains were used and staged under standard conditions as described previously: wild-type ABSR, *Tg(lyz:dsRed)* (Hall et al., 2007) and *c-myb^hkz3^* mutant (Zhang et al., 2011). *Tg(lyz:dsRed)^+/+^*; *c-myb^hkz3/+^* were crossed with *c-myb^hkz3/+^* and the progeny of *c-myb^hkz3/hkz3^* mutants and siblings were separated based on dsRed (dsRed^+^ embryos represented siblings and dsRed^−^ embryos were mutants). Embryos were obtained by natural breeding and maintained at 28±0.5°C for subsequent experiments.

### BQ exposure

BQ (Sigma-Aldrich) was dissolved in 100% dimethyl sulfoxide (DMSO). The stock solution was 40 mmol/l, and the treatment solutions (8–10 μmol/l) were obtained by dilution with egg water. The final DMSO concentration in the control was equal to that in each exposure group. Dechorionated embryos were exposed to the treatment solutions of BQ at 24 h post-fertilization (hpf). Live embryos and malformed embryos were counted every 24 h, and dead embryos were removed from the culture.

### WISH

WISH was performed with antisense digoxigenin-labeled RNA probes based on a standard protocol (Westerfield, 2007). The following probes were used: *lyz*, *mpx*, *l-plastin*, *mfap4*, *rag1*, *βe1-globin* and *c-myb*. For quantification, the relative *rag1^+^* signal areas were analyzed using ImageJ (https://imagej.nih.gov/ij/) software, and signal counts for the other probes were calculated in the CHT region manually under the stereomicroscope (Olympus).

### SB staining

Fixed embryos at 5 dpf were incubated in SB solution (Sigma-Aldrich) and washed, according to standard protocols. The stained neutrophils in the CHT region were counted under a stereomicroscope (Olympus).

### RT-qPCR

Total RNA was extracted from the embryos at 2 dpf using TRIzol reagent (Invitrogen) and reverse transcribed with M-MLV Reverse Transcriptase (Promega) according to the manufacturer’s instructions. Complementary DNA from embryos was used for qPCR using a LightCycler+ 96 machine (Roche).

### Cytological analysis

After adult zebrafish were soaked in 2 μmol/l BQ for 7 days, KM cells were re-suspended in 5% fetal bovine serum, followed by centrifugation at 18 ***g*** for 3 min. The cells were stained as described previously (Liu et al., 2017). Blood cells of KM were calculated manually based on their morphology (Carradice and Lieschke, 2008; Stachura and Traver, 2011).

### BrdU labeling

*Tg(lyz:dsRed)* embryos at 3 dpf were incubated in 10 mM BrdU (Sigma-Aldrich) for 2 h. After BrdU treatment, the embryos were washed and fixed for immunohistochemistry as described previously (Lin et al., 2017). The embryos were stained with mouse anti-BrdU (1:50; 11170376001, Roche), followed by Alexa Fluor goat anti-mouse-488 (1:400; A-11001, Invitrogen) for fluorescent visualization.

### Flow cytometry analysis

*Tg(lyz:GFP)* embryos were collected at 3 dpf, and 100 embryos in each group were pooled together and homogenized by needles and syringes. The homogenized samples were then treated with 0.25% Trypsin-EDTA (25200-072, Gibco) at 30°C for 30 min and the reaction was stopped by adding calcium chloride. Suspended cells were collected by centrifugation (400 ***g*** at 4°C), filtered by 35-μm nylon mesh (352235, FALCON) and finally subjected to flow cytometry analysis (FACS Aria IIIu, BD Biosciences). At least 10,000 events were collected for each sample. The live cells were gated using side scatter-A (SSC-A) (granularity) and forward scatter-A (FSC-A) (size) parameters. Discrimination of aggregates from singlets was performed using forward scatter-W (FSC-W) versus FSC-A, and the cell counts in the GFP^+^ gate were given as the percentages of the total singlet population.
